# The association between quality of life and diabetes: the Bushehr Elderly Health Program

**DOI:** 10.1186/s12877-024-04878-6

**Published:** 2024-03-19

**Authors:** Nekoo Panahi, Mohammad Ahmadi, Marjan Hosseinpour, Amin Sedokani, Mahnaz Sanjari, Kazem Khalagi, Mohammad Javad Mansourzadeh, Akram Farhadi, Iraj Nabipour, Bagher Larijani, Noushin Fahimfar, Afshin Ostovar

**Affiliations:** 1 Metabolic Disorders Research Center, Endocrinology and Metabolism Molecular-Cellular Sciences Institute, Tehran University of Medical Sciences, Tehran, Iran; 2 Endocrinology and Metabolism Research Center, Endocrinology and Metabolism Clinical Sciences Institute, Tehran University of Medical Sciences, Tehran, Iran; 3 Osteoporosis Research Center, Endocrinology and Metabolism Clinical Sciences Institute, Tehran University of Medical Sciences, Tehran, Iran; 4 Department of Epidemiology and Biostatistics, School of Public Health, Tehran University of Medical Sciences, Tehran, Iran; 5 Obesity and Eating Habits Research Center, Endocrinology and Metabolism Clinical Sciences Institute, Tehran University of Medical Sciences, Tehran, Iran; 6 The Persian Gulf Tropical Medicine Research Center, The Persian Gulf Biomedical Sciences Research Institute, Bushehr University of Medical Sciences, Bushehr, Iran; 7 The Persian Gulf Marine Biotechnology Research Center, The Persian Gulf Biomedical Sciences Research Institute, Bushehr University of Medical Sciences, Bushehr, Iran

**Keywords:** Diabetes, Quality of life, SF-12 questionnaire, Aged, Depression

## Abstract

**Background and objective:**

Considering the importance of diabetes and its increased prevalence with aging, this study aimed to evaluate the association between diabetes status and quality of life (QOL) and the determining factors in individuals over 60.

**Methods:**

Two thousand three hundred seventy-five individuals including 819 (34.5%) with diabetes, aged 69.4 ± 6.4, from Bushehr Elderly Health Program (BEHP) were enrolled. We categorized the participants as non-diabetic, controlled diabetic, and poorly controlled diabetic. The QOL was assessed using the SF-12 questionnaire. The physical (PCS) and mental (MCS) component summaries of QOL were estimated. We compared the SF-12 domains and components between the categories using ANOVA. Further, the association of diabetes status with PCS and MCS was assessed after adjustment for possible confounders including age, sex, depression, cognitive impairment, physical activity, and other relevant factors using linear regression analysis.

**Results:**

Individuals with diabetes had lower PCS (40.9 ± 8.8 vs. 42.7 ± 8.6, *p*-value < 0.001), and MCS scores (45.0 ± 10.2 vs. 46.4 ± 9.4, *p*-value < 0.001) compared to participants without diabetes. No significant differences were observed in PCS or MCS scores between controlled or poorly controlled individuals with diabetes. Diabetes status was associated with PCS and MCS scores in univariable analysis. Regarding physical component of QOL, after adjusting for other confounders, poorly controlled diabetes was significantly associated with PCS [beta: -1.27 (-2.02, -0.52)]; some other determinants include depression [-7.66 (-8.51, -6.80)], male sex [3.90 (3.24,4.57)], and good physical activity [1.87 (1.17,2.57)]. As for the mental component, controlled diabetes was significantly associated with MCS [-1.17 (-2.13, -0.22)]; other contributing factors include depression [-14.35 (-15.34, -13.37)], male sex [1.97 (1.20,2.73)], good physical activity [-1.55 (-2.35, -0.75)], and smoking [-1.42 (-2.24, -0.59)]. BMI had an inverse association with PCS [-0.19 (-0.26, -0.13)] and a direct association with MCS [0.14 (0.07,0.21)].

**Conclusion:**

Individuals with diabetes exhibited reduced QOL scores. Upon adjusting for other variables, it was found that uncontrolled diabetes correlated with decreased PCS scores, whereas controlled diabetes was linked to lower MCS scores. Factors such as depression and being female were identified as contributors to diminished QOL in both physical and mental aspects. These results have the potential to guide healthcare decision-making, facilitating the creation of tailored interventions aimed at improving the QOL for individuals with diabetes, with a specific focus on women and depression.

## Introduction

The prevalence of diabetes is on the rise as our population ages, with an estimated 463 million individuals worldwide currently affected by the disease [1]. Diabetes presents unique challenges for older adults, leading to a deterioration of various organs and substantial burdens. These challenges stem from complications such as macrovascular (cardiovascular diseases) and microvascular issues (such as retinopathy, nephropathy, and neuropathy), acute conditions (such as diabetic ketoacidosis, acute myocardial infarction, and a hyperglycemic hyperosmolar state), and heightened risks of morbidity and mortality [2]. Older adults with diabetes are at a greater risk of developing diabetes-related complications such as hypoglycemia, kidney failure, and heart disease than younger individuals are. Patients also often have coexisting conditions, such as cognitive impairment and cardiovascular disease, which impact diabetes education and management. Special considerations should be given to support overall health and quality of life (QOL), and relevant therapies for glycemic control, hyperlipidemia, and hypertension are important in this population. These physical aspects significantly impact QOL for these patients, as do essential psychological and social dimensions, highlighting the critical need for optimal care [3]. Social and psychological components encompass concerns such as diabetes-related distress, which involves constant worry about social support, treatment regimens, medications, interpersonal relationships, and emotional stress, as well as psychiatric comorbidities such as depression, anxiety disorders, and eating disorders [4]. Multiple factors that might contribute to QOL in this population include age, sex, body mass index (BMI), education and employment, marital status, smoking, physical activity, hypertension, diabetes duration and control, type of medication, and psychological well-being [5–8].

Previous research has extensively investigated health-related QOL among individuals with diabetes. Studies have examined various facets of QOL, including physical, mental, and social well-being, shedding light on the multifaceted impact of diabetes on different aspects of patients’ lives. Factors such as disease management, complications, treatment adherence, and the psychosocial effects of living with a chronic condition have been central themes in exploring the dimensions of QOL in diabetic populations [5, 9–12]. However, considering the notable surge in Type 2 Diabetes (T2D)-related Disability-Adjusted Life Years (DALYs) and fatalities in middle-income countries such as Iran, which surpassed other Middle East and North Africa (MENA) countries over the last three decades [13], further investigation in this region is mandatory. Additionally, while numerous studies have explored this topic [14, 15], our focus is specifically on adults aged 60 and above due to the rapid aging of the population. The World Health Organization (WHO) predicts that by 2030, 1 in 6 individuals will be older than 60, and this proportion is projected to increase to approximately 1 in 4 by 2050 [16].

Therefore, we aimed to evaluate the association of diabetes status with QOL in individuals over 60, and identify other determinants in a representative sample of the Iranian elderly population using the SF-12 questionnaire. Given the low proportion of diabetes control in Iran [17], we aimed to assess the effect of diabetes control as well. This research offers valuable understanding of high-risk populations pertaining to diabetes, facilitating the creation of focused strategies and interventions designed to meet the unique requirements of affected individuals. By implementing the SF-12 questionnaire as a screening tool, it becomes possible to identify those at higher risk within older adult demographics. Consequently, these findings pave the way for customized approaches aimed at improving QOL and enhancing functional abilities through targeted intervention programs.

## Methods

### Participants

The present study was conducted in individuals participating in the second stage of the Bushehr Elderly Health (BEH) program. BEHP is a population-based prospective cohort study that was conducted on 3000 elderly women and men aged above 60 years, in Bushehr, a southern province of Iran since 2013. The primary objective of this program was to evaluate the prevalence of non-communicable diseases and extensively explore the associated risk factors and resulting implications. Detailed descriptions of the BEHP’s design and methodologies have been previously documented [18, 19]. The inclusion criteria of BEHP were all men and women aged 60 and above who were residents of Bushehr City, excluding participants with a lack of proper follow-up, serious complications of previous medical diseases, or unwillingness to participate. The 2015 launch of the second stage of the BEHP involved 2772 participants and focused primarily on investigating musculoskeletal diseases, cognitive impairments, and the associated risk factors contributing to these conditions. Ethical approval was obtained from the Ethics Committees at the Endocrinology and Metabolism Research Institute of Tehran University of Medical Sciences and Bushehr University of Medical Sciences, marked with the ethical codes IR.TUMS.EMRI.REC.1394.0036 and B-91–14-2, respectively. All the participants had given an informed consent. Participants of the second stage with accessible data on their diabetes status and QOL (*n* = 2375) were enrolled in the present study.

### Measurements

Overnight fasting venous blood sample was obtained for all participants for biochemical measurement and other clinical examinations were taken according to the standard protocols. Additionally, several other factors were considered, including demographic details such as age, sex, education, and marital status. Medical history, QOL assessments, functional and mental well-being evaluations, and lifestyle behaviors (such as smoking, alcohol consumption, physical activity levels, and nutritional status) were gathered through self-reported information acquired through multiple validated questionnaires administered by trained staff. We used available data encompassing various laboratory tests and clinical parameters, such as fasting blood sugar (FBS), HbA1c, BMI, systolic blood pressure (SBP), and diastolic blood pressure (DBP), medical history, as well as QOL data for each participant.

### Variables

Regarding diabetes status, participants were categorized to 3 groups: non-diabetic, controlled diabetics, poorly controlled diabetics. Diabetes was determined to be positive under specific criteria: laboratory findings meeting the thresholds of fasting blood sugar (FBS) ≥ 126 mg/dl or HbA1c ≥ 6.5% or a documented history of diabetes coupled with the use of antidiabetic medications. Participants diagnosed with diabetes by a physician in either phase one (based on laboratory tests) or phase two (also based on laboratory tests) of the study or those on diabetes medication with a reported prior history of the condition were classified as diabetic. Within diabetic individuals, those with an FBS above 130 or an HbA1c equal to or greater than 7% were categorized as poorly controlled diabetes.

QOL was assessed using the self-reported SF-12 questionnaire and the condensed SF-36 questionnaire. This evaluation comprises eight dimensions, grouped into physical and mental components. The physical component encompasses physical functioning, general health, role-physical, and bodily pain, while the mental aspect includes social functioning, mental health, role-emotional, and vitality [20]. The reliability and validity of the SF-12 questionnaire have been examined in Iran previously, with reported Cronbach alpha for physical and mental component summaries of 0.89 and 0.90, respectively, and an excellent discriminatory ability between subgroups of patients based on demographic and clinical variables [21]

Depression was evaluated using the PHQ-9 questionnaire, where scores ranging from eight to 27 were suggestive of depression. Cognitive impairment was assessed through the Categorical Verbal Fluency Test (CFT) and the Mini-Cog. Individuals exhibiting impaired cognition in either of these tests were classified as having cognitive impairment [19].

Hypertension (HTN) status was deemed positive if any of the following criteria were met: a documented history of HTN alongside the use of antihypertensive medications, an SBP measurement equal to or greater than 140 mmHg, or a DBP measurement equal to or greater than 90 mmHg. Comorbidities were assessed based on a positive medical history of chronic respiratory, liver, or kidney diseases or Parkinson’s disease. Notably, HTN was not categorized as a comorbidity in our study due to its high prevalence in the study population (73%). Individuals were classified as nonsmokers or current smokers based on their smoking status. The participants were considered physically active according to the physical activity level using the cutoff value of 1.6 [22].

### Statistical analysis

Continuous data are presented as the mean ± SD, while categorical variables are expressed as numbers (%). Comparisons between the categories (non-diabetic, controlled, and poorly-controlled diabetic) individuals with and without diabetes were conducted using ANOVA, Mann‒Whitney, or chi-squared tests, depending on the data type.

We used univariate linear regression models to assess the relationship between the physical and mental health QOL scores and diabetes status as well as other variables in the whole study population. These models employed the physical or mental component summary (PCS and MCS) score as the dependent variable. We considered various explanatory variables including age, sex, BMI, Medicare supplement insurance, living arrangements, diabetes control, HTN, depression, cognitive impairment, physical activity, education, current smoking status, and comorbidities. Furthermore, multivariable backward stepwise regression analyses were performed to control for potential risk factors and confounding variables identified with a *p*-value < 0.20 in the univariate analyses. Model fitness was evaluated using goodness-of-fit tests, and the model with the smallest Akaike information criterion was considered to have the best fit. Multicollinearity among variables in the final model was assessed using a variance inflation factor (VIF) with a cutoff value 5.0.

We performed the above-mentioned linear regression analysis separately, in diabetic individuals as well to evaluate the association between diabetes control and QOL in diabetics, and to identify the determining factors of PCS and MCS in elderly individuals with diabetes. Despite our efforts to correct potential confounders, it is essential to acknowledge the possibility of unmeasured or residual confounding. Therefore, when interpreting our results, we considered the potential impact of confounding factors.

STATA and Microsoft Excel software were utilized for data analysis, and a *p*-value of 0.05 was considered to indicate statistical significance.

## Results

### Participants characteristics

Of the 2375 participants, 819 (34.5%) had diabetes. Among those with diabetes, 466 (56.9%) had poorly controlled diabetes. Table 1 outlines the characteristics of the participants.

**Table 1:** Characteristics of the study participants

Characteristics	All (*N* = 2375)	Diabetes			*P*-Value^3^	*P*-Value^4^
		Yes (*N* = 819)		No (*N* = 1556)		
		Poorly controlled (*N* = 466)	Well controlled (*N* = 353)			
**Age, years**^**1**^	69.36 (6.41)	68.30 (5.33)	69.20 (6.34)	69.72 (6.69)	< 0.001	0.027
**Body mass index, kg/m2**^**1**^	27.52 (4.90)	28.42 (5.09)	28.66 (4.92)	26.99 (4.76)	< 0.001	0.487
**Sex, male (%)**	1146 (48.25)	212 (45.49)	147 (41.64)	787 (50.58)	0.002	0.271
**Living alone**	119 (5.01)	23 (4.94)	18 (5.10)	78 (5.02)	0.992	0.915
**Medicare supplement insurance**	1419 (59.75)	278 (59.66)	215 (60 .91)	926 (59.51)	0.747	0.717
**Hypertension**	1735 (73.05)	369 (79.18)	280 (79.32)	1086 (69.79)	< 0.001	0.962
**Depression**	329 (13.85)	76 (16.31)	57 (16.15)	196 (12.60)	0.047	0.988
**Cognitive impairment**	1419 (59.75)	300 (64.38)	221 (62.61)	898 (57.71)	0.005	0.602
**Physical activity**	543 (22.86)	98 (21.03)	61 (17.28)	384 (24.68)	0.004	0.179
**Education, years**	5 (0- 9)	4 (0- 7)	4 (0- 9)	5 (0- 9)	0.023^(2)^	0.550
**Current smoker**	491 (20.67)	82 (17.60)	63 (17.85)	346 (22.24)	0.010	0.926
**Comorbidity**	1353 (56.97)	288 (61.80)	165 (46.74)	900 (57.84)	0.237	< 0.001

Mean (SD) for age and BMI, median (Q1-Q3) for education, and number (%) for other variables are presented

^1^Anova

^2^Mann-Whitney, and chi-squared tests were used

^3^*P*-values for comparison between non-diabetic and diabetic patients

^4^in diabetic subgroups

The mean age was significantly lower in the diabetic group, and within the diabetic patients, it was lower among those with poorly controlled diabetes (*p* values < 0.001 and 0.03, respectively). BMI was greater in diabetic patients overall, but there was no significant difference between the subgroups of diabetes (*p* values < 0.001 and 0.5, respectively). A greater proportion of diabetic patients were women than non-diabetic patients were. However, there was no difference within the diabetic subgroups (*p*-values of 0.002 and 0.3, respectively). Hypertension, depression, and cognitive impairment were more common in diabetic patients (*p* values < 0.001, 0.05, and 0.005, respectively), while good physical activity and smoking were less common in participants with diabetes (*p* values: 0.004 and 0.01, respectively). There was no significant difference between the diabetic subgroups regarding these variables. However, comorbidities were significantly more common in those with poorly controlled diabetes than in those with controlled diabetes (61.8% vs. 46.7%, *p* value < 0.001).

### QOL domains and components in the study participants

Individuals without diabetes had significantly greater scores for all the components as presented in detail in Table 2. However, no significant difference was observed between controlled and poorly-controlled diabetic participants in this respect.

**Table 2:** QOL domains and components of the study participants

QOL assessed by SF-12	Diabetes			*P*-Value^1^	*P*-Value^2^
	Yes		No (*N* = 1556)		
	Poorly controlled (*N* = 466)	Well controlled (*N* = 353)			
**Components**					
**Physical Functioning (PF)**	43.12 (13.58)	42.49 (13.28)	45.01 (13.02)	< 0.001	0.51
**Role Physical (RP)**	25.85 (4.51)	26.28 (4.40)	26.74 (4.22)	< 0.001	0.17
**Bodily Pain (BP)**	50.92 (11.62)	50.89 (11.53)	52.74 (9.99)	< 0.001	0.96
**General Health (GH)**	36.53 (10.65)	37.81 (10.40)	39.73 (10.71)	< 0.001	0.08
**Vitality (VT)**	56.64 (12.98)	56.64 (12.46)	58.60 (11.97)	< 0.001	0.99
**Social Functioning (SF)**	51.64 (10.57)	51.16 (10.65)	52.30 (9.62)	0.04	0.51
**Role Emotional (RE)**	18.71 (5.30)	18.44 (5.39)	19.45 (4.96)	< 0.001	0.47
**Mental Health (MH)**	54.22 (13.32)	54.09 (13.44)	56.32 (12.10)	< 0.001	0.89
**Scales**					
**PCS-12**					
**Mean (SD)**	40.79 (8.93)	41.07 (8.71)	42.73 (8.6)	< 0.001	0.65
**Median (Q1–Q3)**	41.85 (34.56–48.86)	42.90 (34.56–49.13)	45.37 (36.55–49.13)		
**MCS–12**					
**Mean (SD)**	45.15 (10.02)	44.91 (10.50)	46.36 (9.41)	< 0.001	0.74
**Median (Q1–Q3)**	48.59 (40.06–51.74)	48.75 (38.19–51.74)	50.95 (42.01–51.74)		

*QOL* quality of life, *QOL* domains and components assessed by SF-12; Mean (SD) is presented for the components, *PCS* Physical Component Summary, *MCS* Mental Component Summary

^1^*P*-values for comparison between non-diabetic and diabetic patients

^2^in diabetic subgroups

The mean PCS-12 scores were 42.73 ± 8.6, 41.07 ± 8.71, and 40.79 ± 8.93 in the non-diabetic, well-controlled diabetic, and poorly controlled diabetic groups, respectively. The respected scores for the MCS-12 were 46.36 ± 9.41, 44.91 ± 10.50, and 45.15 ± 10.02. A significantly lower mean scores was evident in diabetic individuals (*p*-value < 0.001). However, the difference was not significant between the diabetic groups.

### Association between diabetes status and QOL in the elderly individuals

#### PCS

Diabetes status was associated with PCS scores in the univariate regression analysis [beta: -1.65, 95%CI (-2.66, -0.65) for controlled diabetes, and -1.93(-2.83, -1.03) for poorly controlled diabetes], as demonstrated in Table 3. Other factors directly associated with PCS scores in the univariate analysis include male sex, Medicare supplement insurance, good physical activity, years of education, and smoking. Age, BMI, living alone, hypertension, depression, cognitive impairment, and comorbidities were negatively associated PCS scores. In the multivariable regression analysis, after adjusting for the confounders, poorly controlled diabetes association with PCS scores remained significant [-1.27 (-2.02, -0.52)]. Regarding other determinants of PCS, male sex and good physical activity had the strongest positive associations [(3.90 (3.24,4.57) and 1.87 (1.17,2.57), respectively], followed by smoking, and years of education. Conversely, depression exhibited the highest negative association [-7.66 (-8.51, -6.80)], followed by age and BMI. Cognitive impairment showed borderline association.

**Table 3:** The association of diabetes state with physical and mental scales of QOL in the total study population

	Physical Component Summary (PCS)			
Variables	Crude beta coefficient (95% CI)	*P*-value	Adjusted beta coefficient (95% CI)	*P*-value
**State of diabetes, non-diabetic**	(Ref)	_	(Ref)	_
**Good control**	-1.65 (-2.66,-0.65)	0.001	-0.63 (-1.46,0.19)	0.136
**Poor control**	-1.93 (-2.83, -1.03)	< 0.001	-1.27 (-2.02,-0.52)	0.001
**Age, years**	-0.34 (-0.39,-0.28)	< 0.001	-0.28 (-0.33,-0.24)	< 0.001
**Sex, male**	6.55 (5.90,7.20)	< 0.001	3.90 (3.24,4.57)	< 0.001
**Body mass index, kg/m2**	-0.29 (-0.36,-0.22)	< 0.001	-0.19 (-0.26,-0.13)	< 0.001
**Living alone, yes**	-1.72 (-3.33,-0.11)	0.035	_	0.603
**Medicare supplement insurance, yes**	2.00 (1.28,2.71)	< 0.001	_	0.666
**Hypertension, yes**	-1.92 (-2.71,-1.13)	< 0.001	_	0.457
**Depression, yes**	-10.18 (-11.11,-9.25)	< 0.001	-7.66 (-8.51,-6.80)	< 0.001
**Cognitive impairment, yes**	-3.45 (-4.15,-2.75)	< 0.001	-0.63 (-1.26,0.01)	0.052
**Physical activity, good**	3.44 (2.61,4.26)	< 0.001	1.87 (1.17,2.57)	< 0.001
**Education, years**	0.57 (0.50,0.64)	< 0.001	0.19 (0.12,0.26)	< 0.001
**Current smoker, yes**	1.08 (0.21,1.95)	0.014	0.87 (0.15,1.59)	0.017
**Comorbidity, yes**	-1.16 (-1.87,-0.45)	0.001	_	0.660
	**Mental Component Summary (MCS)**			
**Variables**	**Crude beta coefficient (95% CI)**	***P*-value**	**Adjusted beta coefficient (95% CI)**	***P*-value**
**State of diabetes, non-diabetic**	_	_	_	_
**Good control**	-1.44 (-2.56,-0.32)	0.011	-1.17 (-2.13,-0.22)	0.016
**Poor control**	-1.21 (-2.21,-0.20)	0.018	-0.83 (-1.68,0.02)	0.058
**Age, years**	0.08 (0.02,0.14)	0.004	0.10 (0.05,0.16)	< 0.001
**Sex, male**	3.68 (2.91,4.44)	< 0.001	1.97 (1.20,2.73)	< 0.001
**Body mass index, kg/m2**	-0.01 (-0.08,0.07)	0.971	0.14 (0.07,0.21)	< 0.001
**Living alone, yes**	-0.24 (-2.04, 1.54)	0.784	_	0.627
**Medicare supplement insurance, yes**	1.42 (0.63,2.22)	< 0.001	0.64 (-0.06,1.35)	0.074
**Hypertension, yes**	-0.27 (-1.15,0.60)	0.537	_	0.355
**Depression, yes**	-14.64 (-15.61,-13.68)	< 0.001	-14.35 (-15.34,-13.37)	< 0.001
**Cognitive impairment, yes**	-1.10 (-1.90,-0.31)	0.006	_	0.593
**Physical activity, good**	-0.96 (-1.89,-0.03)	0.041	-1.55 (-2.35,-0.75)	< 0.001
**Education, years**	0.11 (0.04,0.19)	0.002	-0.08 (-0.16,-0.008)	0.030
**Current smoker, yes**	-1.94 (-2.91,-0.98)	< 0.001	-1.42 (-2.24,-0.59)	0.001
**Comorbidity, yes**	-0.81 (-1.60,-0.02)	0.042	_	0.822

*QOL* quality of life, *QOL* components assessed by SF-12; univariable and multivariable regression analysis

#### MCS

Diabetes status was associated with MCS scores in the univariate regression analysis [-1.44 (-2.56, -0.32) for controlled diabetes, and -1.21 (-2.21, -0.20)] for poorly controlled diabetes], as demonstrated in Table 3. Age, male sex, Medicare supplement insurance, and years of education were positively, and depression, cognitive impairment, good physical activity, and smoking were negatively associated with MCS scores in the univariate regression analysis. After accounting for confounding variables in the multivariable regression analysis, controlled diabetes continued to show a significant association with MCS scores [-1.17 (-2.13, -0.22)]; however, the association of poorly controlled diabetes were marginal [-0.83 (-1.68,0.02)]. Regarding other determinants of MCS, male sex had the strongest positive association [1.97 (1.20,2.73)], followed by BMI [0.14 (0.07,0.21)], and age [0.10 (0.05,0.16)]. Conversely, depression showed the highest negative association [-14.35 (-15.34, -13.37)], followed by good physical activity [-1.55 (-2.35, -0.75)], smoking [-1.42 (-2.24, -0.59)], and years of education [-0.08 (-0.16, -0.008)].

### Association between diabetes control and QOL in elderly diabetic individuals

Diabetes control was not associated with PCS and MCS scores among the 819 elderly individuals with diabetes in the univariable and multivariable regression analysis. Several determining factors of physical and mental components of QOL in individuals with diabetes are presented in Table 4. In the multivariable analysis, male sex [4.25, (3.10 to 5.39)], good physical activity [2.30 (1.04, 3.56)], and years of education [0.22 (0.11, 0.33)] were positively associated with PCS scores. Depression [-8.01 (-9.37, -6.64)], BMI [-0.23 (-0.33, -0.13)], and age [-0.22 (-0.31, -0.13)] were inversely associated with PCS. Regarding the MCS, different associations were observed. While male sex [1.5 (0.25, 2.81)], age [0.15 (0.04, 0.25)], and BMI [0.12 (0.003, 0.25)] were positively associated with MCS, depression showed a significant inverse association [-13.96 (-15.63, -12.28)]. Physical activity and smoking were not significantly associated with MCS scores in individuals with diabetes.

**Table 4:** The association of diabetes control with physical and mental scales of QOL in the diabetic individuals

	Physical Component Summary (PCS)			
Variables	Crude beta coefficient (95% CI)	*P*-value	Adjusted beta coefficient (95% CI)	*P*-value
**Control of diabetes, poor control**	-0.27 (-1.50,0.94)	0.654	_	0.209
**Age, years**	-0.26 (-0.37,-0.16)	< 0.001	-0.22 (-0.31,-0.13)	< 0.001
**Sex, male**	7.12 (6.00,8.24)	< 0.001	4.25 (3.10,5.39)	< 0.001
**Body mass index, kg/m2**	-0.39 (-0.51,-0.27)	< 0.001	-0.23 (-0.33, -0.13)	< 0.001
**Living alone, yes**	-3.40 (-6.18,-0.63)	0.016	_	0.353
**Medicare supplement insurance, yes**	1.57 (0.34,2.80)	0.012	_	0.680
**Hypertension, yes**	-1.50 (-3.00,-0.01)	0.048	_	0.252
**Depression, yes**	-10.29 (-11.78,-8.81)	< 0.001	-8.01 (-9.37,-6.64)	< 0.001
**Cognitive impairment, yes**	-3.04 (-4.28,-1.79)	< 0.001	_	0.490
**Physical activity, good**	4.15 (2.65,5.66)	< 0.001	2.30 (1.04,3.56)	< 0.001
**Education, years**	0.59 (0.47,0.70)	< 0.001	0.22 (0.10,0.33)	< 0.001
**Current smoker, yes**	0.72 (-0.85,2.31)	0.368	1.02 (-0.26,2.31)	0.119
**Comorbidity, yes**	-1.73 (-2.94,-0.51)	0.005	_	0.726
	**Mental Component Summary (MCS)**			
**Variables**	**Crude beta coefficient (95% CI)**	***P*-value**	**Adjusted beta coefficient (95% CI)**	***P*-value**
**Control of diabetes, poor control**	0.23 (-1.18,1.65)	0.743	_	0.545
**Age, years**	0.14 (0.02,0.26)	0.015	0.15 (0.04,0.25)	0.005
**Sex, male**	3.57 (2.18,4.97)	< 0.001	1.5 (0.25,2.81)	0.019
**Body mass index, kg/m2**	-0.01 (-0.15, 0.12)	0.799	0.12 (0.003,0.25)	0.044
**Living alone, yes**	-0.95 (-4.17,2.25)	0.559	_	0.895
**Medicare supplement insurance, yes**	1.40 (-0.02,2.83)	0.055	_	0.776
**Hypertension, yes**	-0.23 (-1.96,1.49)	0.787	_	0.859
**Depression, yes**	-14.12 (-15.77,-12,48)	< 0.001	-13.96 (-15.63,-12.28)	< 0.001
**Cognitive impairment, yes**	-0.88 (-2.34,0.57)	0.233	_	0.791
**Physical activity, good**	-0.55 (-2.33,1.21)	0.536	-1.43 (-2.97,0.11)	0.070
**Education, years**	0.13 (-0.01,0.27)	0.066	_	0.364
**Current smoker, yes**	-1.99 (-3.83,-0.16)	0.033	-1.19 (-2.77,0.37)	0.135
**Comorbidity, yes**	-0.82 (-2.23,0.58)	0.253	_	0.795

*QOL* quality of life, *QOL* components assessed by SF-12; univariable and multivariable regression analysis

Overall, depression was negatively associated with both PCS and MCS scores, and male sex was positively associated with both PCS and MCS scores in diabetic individuals. However, age and BMI showed discrete associations. These factors were negatively associated with the PCS score and positively associated with the MCS score (Table 4).

## Discussion

Our research included a sample of 2375 participants to evaluate QOL among the participants regarding their diabetes status. Our analysis revealed significantly diminished health-related QOL scores across all SF-12 components among diabetic participants compared to their non-diabetic counterparts. However, when inspecting the scores among diabetic subgroups (well-controlled and poorly controlled), no statistically significant variance in SF-12 component scores emerged. Both the mean MCS-12 score and mean PCS-12 score were notably lower in individuals with diabetes than in those without diabetes. Nevertheless, these scores exhibited no significant differences within diabetic subgroups. Depression, sex, age, BMI, physical activity, and education were found to be determinants of QOL in our study population.

Lower PCS and MCS scores in participants with diabetes suggest a substantial influence of diabetes on both the physical and mental health domains. Poorly controlled diabetes was an independent determinant of lower PCS scores in our study population of elderly individuals. Our findings corroborate earlier studies conducted in diverse geographical settings. A recent meta-analysis underscored lower QOL among individuals with diabetes than among the general population. Additionally, a cross-sectional study conducted in Saudi Arabia corroborated a significantly reduced health-related QOL among patients with diabetes compared to controls [23]. Another study indicated that, compared to individuals in the control group, individuals with type 2 diabetes registered a decrease ranging from 11 to 27% across six SF-20 components. Additionally, the mean scores for both the PCS and MCS were notably lower among these patients, significantly impacting their physical and mental health dimensions [24]. In contrast, findings from a study conducted in India diverged from our research outcomes. Their study reported an absence of a statistically significant disparity in QOL scores between individuals with and without diabetes [25]. Some studies have reported better mental health in patients with well-controlled diabetes, one of the few components of QOL that improve after intervention [26, 27]; however, we observed that controlled diabetes was associated with lower MCS scores. Long-term poor control can lead to complications such as neuropathy, nephropathy, retinopathy, and peripheral arterial disease, which can impair QOL. We found no significant association between diabetes control and QOL scores among diabetic individuals considering other contributing factors. This could be partly due to our lack of data on disease duration. Additionally, the participants of the BEHP were those with adequate physical and mental ability to participate in the program, and those with severe complications were not included initially.

In our investigation of factors linked to health-related QOL among our participants, we identified several influential associations. Male sex and regular physical activity were the most impactful positive factors contributing to higher PCS scores. Conversely, depression was identified as the most substantial negative contributor to PCS scores. Moreover, age and BMI displayed negative associations with the PCS score and positive associations with the MCS score that remained significant after we adjusted for possible confounders. The adverse effects of age on the PCS score were demonstrated in previous studies [28, 29]. This finding suggests a trend in which younger participants experience better physical health status than their older counterparts and urges further specific investigations. The decline in physical function and cognitive impairment with advancing age may contribute to the lower PCS scores.

Among the factors associated with MCS scores, male sex was the most influential factor. Patients who possessed Medicare supplement insurance also exhibited marginally higher MCS scores. Age was positively associated with the MCS score. The positive association between age and MCS score is consistent with a study conducted in Egypt, which reported an increase in the score in the psychological domain with age [30]. Conversely, depression displayed a substantial association with a lower MCS score. Additionally, individuals who were current smokers had significantly lower MCS scores. Previous research has highlighted the potential for smoking and alcohol consumption to create feelings of social stigma among patients, potentially contributing to diminished health-related QOL. This finding suggested that such social factors could play a role in lowering overall QOL among affected individuals [31–33].

Being a man emerged as the most influential and independent positive factor for PCS and MCS. Previous studies have consistently reported lower health-related QOL among women than men [23, 34, 35]. However, findings contradicting this trend indicate no significant difference in QOL between men and women with diabetes [36]. Previous research has highlighted that women tend to experience higher rates of obesity and more severe disease conditions. Additionally, they often bear the weight of extra societal and familial responsibilities [9].

Depression emerged as a substantial negative factor associated with significantly lower scores on both the PCS and MCS. This finding aligns logically with extensive studies showcasing the evident impact of depression on the physical and mental well-being of patients [37]. According to a study conducted in the USA that applied a health utility index tool and revealed that patients with multiple comorbidities were more susceptible to experiencing diminished QOL. Moreover, addressing depression and preventing complications are among the most effective strategies for enhancing health-related QOL in individuals with diabetes [38]. In our research sample, we found a link between cognitive impairment and other health conditions with reduced health-related QOL in the initial analysis. Yet, upon accounting for additional factors, this association lost significance. This may show that depression had a more pronounced influence compared to other conditions. Additionally, it is worth noting that participants in the BEHP study possessed sufficient physical and mental capabilities to engage in the program.

Regional cultural and socioeconomic influences are pivotal in shaping the QOL impact [39]. The World Health Organization (WHO) defines QOL as individuals’ perceptions of their life circumstances within the cultural and value frameworks of their environment, in relation to their aspirations, expectations, norms, and worries [40]. Despite this, the influence of cultural disparities on QOL among diabetic individuals remains unexplored. Inadequate self-care practices have been identified as obstacles to the effective management of diabetes and its complications, attributed to factors such as limited access to healthcare facilities and professionals, strained patient-provider relationships, and disruptions in care continuity [41]. Therefore, further prospective studies are warranted to investigate the interplay between diabetes and QOL. The insights gained from such research can be particularly valuable in the Middle East and North Africa (MENA) region due to shared cultural backgrounds and similarities in various health conditions.

In essence, the research seeks to uncover the reasons behind reduced QOL in elderly individuals concerning their diabetes status and considering several contributing factors. The goal is to offer valuable information for healthcare interventions and tactics to enhance QOL among this group. This involves pinpointing factors that can be changed through interventions, like tackling depression, encouraging physical activity, and strengthening social support systems. Additionally, there is a focus on addressing the specific needs of women who tend to experience lower quality of life scores.

## Limitations and strengths

This research has several limitations. First, because of its cross-sectional design, causality cannot be established. Second, the lack of longitudinal data on the duration of diabetes may restrict the ability to determine the impact of poorly controlled diabetes on QOL, as long-standing poor control can lead to complications that further reduce QOL. In addition, we could not assess the effect of the specific type of medication on QOL at this stage. However, our study is distinctive because it is one of the few inquiries into health-related QOL and the determining factors in elderly individuals considering their diabetes status in the region, and it boasts a substantial sample size. The insights from this research can potentially provide valuable data for clinicians, health policymakers, and social workers.

## Conclusion

The study revealed that elderly individuals diagnosed with diabetes have reduced QOL scores compared to those without diabetes. Factors such as depression and being female were notably linked to lower physical and mental QOL aspects. Furthermore, age and BMI showed a negative correlation with PCS and a positive correlation with MCS. Engaging in physical activity and having more years of education were positively associated with PCS and negatively associated with MCS. Notably, no significant relationship was found between diabetes management and QOL among participants with diabetes, potentially due to insufficient data on diabetes duration. These insights can be valuable for healthcare decision-making and the creation of targeted interventions to improve the QOL of elderly individuals, particularly focusing on women and individuals dealing with depression.

## Data Availability

The datasets used and/or analysed during the current study are available from the corresponding author on reasonable request.
