# Correction: The association between quality of life and diabetes: the Bushehr Elderly Health Program

**DOI:** 10.1186/s12877-024-04921-6

**Published:** 2024-05-24

**Authors:** Nekoo Panahi, Mohammad Ahmadi, Marjan Hosseinpour, Amin Sedokani, Mahnaz Sanjari, Kazem Khalagi, Mohammad Javad Mansourzadeh, Akram Farhadi, Iraj Nabipour, Bagher Larijani, Noushin Fahimfar, Afshin Ostovar

**Affiliations:** 1 Metabolic Disorders Research Center, Endocrinology and Metabolism Molecular-Cellular Sciences Institute, Tehran University of Medical Sciences, Tehran, Iran; 2 Endocrinology and Metabolism Research Center, Endocrinology and Metabolism Clinical Sciences Institute, Tehran University of Medical Sciences, Tehran, Iran; 3 Osteoporosis Research Center, Endocrinology and Metabolism Clinical Sciences Institute, Tehran University of Medical Sciences, Tehran, Iran; 4 Department of Epidemiology and Biostatistics, School of Public Health, Tehran University of Medical Sciences, Tehran, Iran; 5 Obesity and Eating Habits Research Center, Endocrinology and Metabolism Clinical Sciences Institute, Tehran University of Medical Sciences, Tehran, Iran; 6 The Persian Gulf Tropical Medicine Research Center, The Persian Gulf Biomedical Sciences Research Institute, Bushehr University of Medical Sciences, Bushehr, Iran; 7 The Persian Gulf Marine Biotechnology Research Center, The Persian Gulf Biomedical Sciences Research Institute, Bushehr University of Medical Sciences, Bushehr, Iran

**Correction: BMC Geriatrics (2024) 24:267**

10.1186/s12877-024-04878-6

After publication of this article, the authors reported that the author name ‘Mohammad Ahmadi’ was incorrectly written as ‘Mohamad Ahmadi’, and the author name ‘Mohammad Javad Mansourzadeh’ was incorrectly written as ‘Mohamad Javad Mansourzadeh’.

The original article has been updated by the authors.

